# Structural basis for sequence-specific recognition of guide and target strands by the *Archaeoglobus fulgidus* Argonaute protein

**DOI:** 10.1038/s41598-023-32600-w

**Published:** 2023-04-14

**Authors:** Elena Manakova, Edvardas Golovinas, Reda Pocevičiūtė, Giedrius Sasnauskas, Algirdas Grybauskas, Saulius Gražulis, Mindaugas Zaremba

**Affiliations:** Life Sciences Center, Institute of Biotechnology, Vilnius University, Sauletekio Av. 7, 10257 Vilnius, Lithuania

**Keywords:** DNA, Enzymes, RNA, Structural biology

## Abstract

Argonaute (Ago) proteins are found in all three domains of life. The best-characterized group is eukaryotic Argonautes (eAgos). Being the structural core of RNA interference machinery, they use guide RNA molecules for RNA targeting. Prokaryotic Argonautes (pAgos) are more diverse, both in terms of structure (there are eAgo-like ‘long’ and truncated ‘short’ pAgos) and mechanism, as many pAgos are specific for DNA, not RNA guide and/or target strands. Some long pAgos act as antiviral defence systems. Their defensive role was recently demonstrated for short pAgo-encoding systems SPARTA and GsSir2/Ago, but the function and action mechanisms of all other short pAgos remain unknown. In this work, we focus on the guide and target strand preferences of AfAgo, a truncated long-B Argonaute protein encoded by an archaeon *Archaeoglobus fulgidus*. We demonstrate that AfAgo associates with small RNA molecules carrying 5′-terminal AUU nucleotides in vivo, and characterize its affinity to various RNA and DNA guide/target strands in vitro. We also present X-ray structures of AfAgo bound to oligoduplex DNAs that provide atomic details for base-specific AfAgo interactions with both guide and target strands. Our findings broaden the range of currently known Argonaute-nucleic acid recognition mechanisms.

## Introduction

Argonaute (Ago) proteins are found in all three domains of life (bacteria, archaea, and eukaryotes). The best-characterized group is eukaryotic Ago (eAgo) proteins. Being the functional core of RNA interference machinery, eAgos are involved in the regulation of gene expression, silencing of mobile genome elements, and defence against viruses^1,2^. From the structural and mechanistic point of view, all characterized eAgos are very similar, as they all use small (~ 13–30 nt) RNA molecules as guides for sequence-specific recognition of RNA targets, and are monomeric proteins sharing four major conserved functional domains and two linker domains, which are organized in a bilobed structure^3–5^. The N-terminal lobe consists of N-domain that separates guide and target strands^6^, and PAZ domain responsible for binding the 3′-terminus of the guide RNA; the C-terminal lobe consists of MID domain, which binds the 5′-terminus of the guide RNA, and PIWI domain, an RNase H homologue^1,2,7,8^. The two linker domains, L1 and L2 connect N and PAZ and PAZ and MID domains, respectively. Upon recognition of the RNA target, eAgos may either cleave it employing catalytic activity of the PIWI domain or, particularly eAgo proteins that encode catalytically inactive PIWI domains, recruit partner proteins leading to degradation of the target RNA or repression of its translation^1,9^. Target specificity of eAgos is determined solely by correct base pairing between guide and target RNA strands. Nevertheless, all eAgos associate only with 5′-phosphorylated guide RNAs, and many have intrinsic specificity for the 5′-terminal nucleotide of the guide RNA (gRNA). For example, *A. thaliana* AGO1, *K. polysporus* KpAgo, and *H. sapiens* hAgo2 prefer guide RNAs with a 5′-terminal uridine, *A. thaliana* AGO2 prefers 5′-A, and *A. thaliana* AGO5 prefers 5′-C^10–13^. The 5′-phosphate and the 5′-terminal nucleotide are recognized in conserved pockets of the MID domain.

Ago proteins are also identified in 9% and 32% of sequenced bacterial and archaeal genomes, respectively^14^. Unlike eAgos, prokaryotic Agos (pAgos) are diverse in terms of their structure, mechanism, and function^8,15–18^. The best understood are the so-called full-length or long pAgos, which are composed of N, PAZ, MID and PIWI domains, and thus closely resemble eAgos. There is mounting evidence that long pAgos function as prokaryotic antiviral systems, with the PIWI domain performing cleavage of invading nucleic acids^1,13^. However, unlike eAgos, which canonically use RNA guides for recognition of RNA targets in a process called RNA interference (RNAi)^19–21^, different long pAgos may use either RNA or DNA guides and/or targets^13,14,18^, and in vitro may associate with phosphorylated (e.g. AaAgo, PfAgo, and RsAgo from *Aquifex aeolicus, Pyrococcus furiosus,* and *Rhodobacter sphaeroides*, respectively)^13^, non-phosphorylated guide strands (e.g., MpAgo and TpAgo from *Marinitoga piezophila* and *Thermotoga profunda*, respectively) or lack preference for 5′-phosphorylation (CbAgo, LrAgo, KjAgo from *Clostridium butyricum*, *Limnothrix rosea*, *Kordia jejudonensis*, respectively)^13,22–24^. Interestingly, the recently described KmAgo from *Kurthia massiliensis* can utilize both DNA and RNA guides to cleave DNA and RNA targets in vitro, albeit with different efficiencies^25,26^. Some long pAgos also specifically recognize the 5′-terminal nucleotide of the guide strand, e.g., CbAgo from *Clostridium butyricum* prefers 5′-terminal deoxyadenosine, and TtAgo prefers 5′-terminal dC^13,27^, while RsAgo from *Rhodobacter sphaeroides* prefers guide RNA with 5′-U^28,29^. The majority (~ 60%) of identified pAgos are ‘short’, as they encode just MID and PIWI domains, the latter being catalytically inactive due to active site mutations. The mechanism, guide/target preferences and function of short pAgos is an emerging topic in the Argonaute field, as evidenced by recent characterization of SPARTA and GsSir2/Ago antiviral systems^15,16,30^.

In this work we focus on the truncated long-B^8^ prokaryotic Argonaute AfAgo (also known as AfPIWI or Af1318^31,32^) encoded by a hyperthermophilic archaeon *Archaeoglobus fulgidus*^8^. Although phylogenetically classified as a truncated long-B pAgo, AfAgo contains only MID and catalytically inactive PIWI domains in a single polypeptide chain, akin to typical short pAgos, and could therefore be considered a pseudo-short pAgo^33^. Even though AfAgo is one of the first and one of the best structurally characterized prokaryotic Argonautes, with an apo-, DNA-, and two RNA-bound structures currently available^31,32,34,35^, its guide/target preferences remain undefined. We show here that AfAgo co-purifies with small RNA molecules carrying 5′-terminal AU nucleotides in vivo and investigate its affinity to various RNA and DNA guide/target strands in vitro. We also present X-ray structures of AfAgo bound to DNA oligoduplexes with 5′-ATT and 5′-ATC terminal sequences that provide structural details on the base-specific AfAgo interactions with the termini of both guide and target strands. Our findings broaden the range of currently known Argonaute-nucleic acid recognition mechanisms.

## Results

### Analysis of in vivo AfAgo-bound nucleic acids

**Figure 1:**
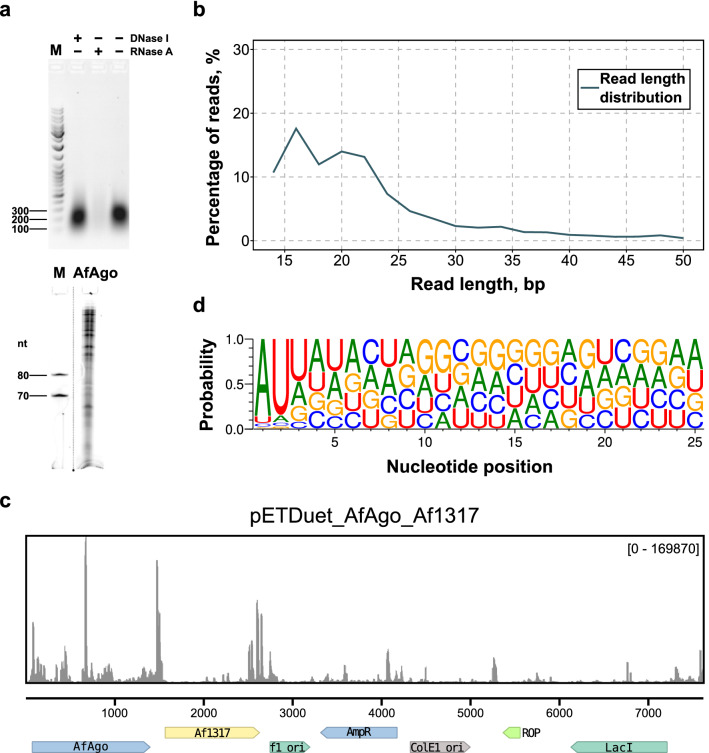
Analysis of in vivo (*E. coli*) AfAgo-bound nucleic acids. (**a**) Top-Digestion of AfAgo nucleic acids with DNAse I and RNase A. Bottom-Size analysis of AfAgo-bound RNA. (**b**) Read length distribution of sequenced AfAgo-bound nucleic acids. (**c**) Sequencing read alignments to the AfAgo expression vector. 73% of all reads map to the expression vector, compared to 27% to *E*. *coli* genome (Supplementary file 1). (**d**) Small RNAs copurified with AfAgo show 5′-AUU bias.

When overexpressed in *E. coli*, AfAgo co-purifies with tightly bound nucleic acids, predominantly RNA (Fig. 1A, Supplementary Fig. S1). This interaction is disrupted only at NaCl concentrations exceeding 1.0 M, implying tight association. The length of the AfAgo-bound RNA varies from a few dozen to a few hundred nucleotides (Fig. 1A), with sequencing data showing that most reads fall between 14 and 30 nucleotides (Fig. 1B). Sequencing of AfAgo-bound RNA revealed that most successfully mapped RNAs (73%) are derived from the AfAgo expression vector (Fig. 1C), while a smaller fraction (27%) was derived from the *E. coli* genome (Supplementary file 1). Surprisingly, AfAgo had a strong preference for two 5′-terminal RNA nucleotides, A at the first and U at the second position (occupancies 0.862 and 0.846, respectively), and a discernible preference for U at the third position (occupancy 0.476, Fig. 1D). Thus, AfAgo, like many eAgos and long pAgos, has intrinsic specificity for the 5′-terminus of the bound nucleic acid.

### AfAgo interactions with nucleic acids in vitro

**Figure 2:**
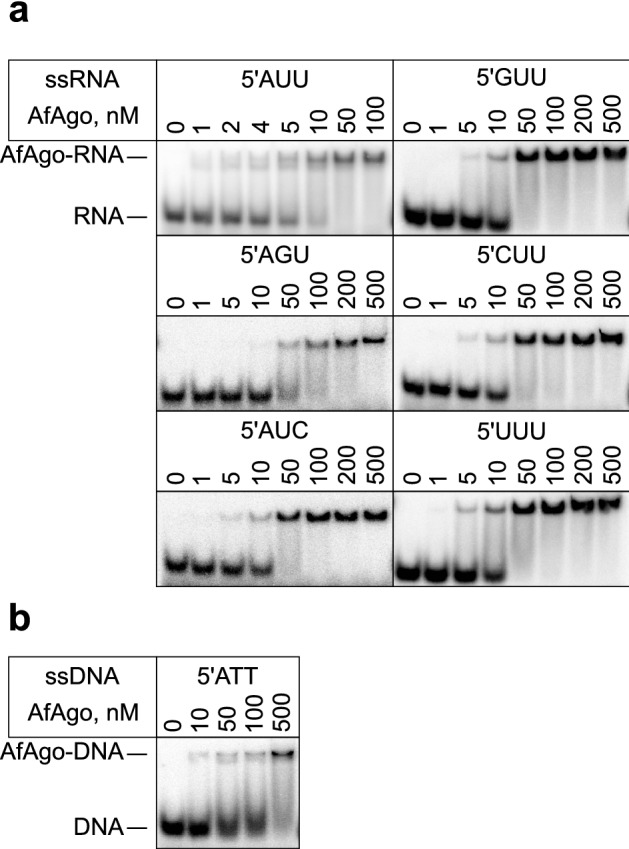
AfAgo interactions with nucleic acids in vitro. EMSA experiments were performed with 5 nM total 5′P-ssRNA (**a**) and ssDNA (**b**), and varying concentrations of AfAgo, indicated above each lane. Calculated K_d_ values are provided in Table 1.

Previous studies suggested that AfAgo has a strong preference for single- and double-stranded DNA over single- or double-stranded RNA^34^. However, these studies were performed using double-stranded DNA with 5′-C and 5′-T terminal nucleotides, neither of which, according to our analysis of in vivo-bound nucleic acids, is optimal for AfAgo binding. To re-evaluate AfAgo affinity to nucleic acids, we have employed the electrophoretic mobility shift assay (EMSA) and synthetic single-stranded (ss) RNA and DNA oligonucleotides containing phosphorylated 5′-AUU and 5′-ATT termini, respectively (Supplementary Table S1). The experiments revealed that under our experimental conditions (see Methods), AfAgo preferentially binds ssRNA over ssDNA. Binding of ssDNA was detected only at exceedingly high (> 0.5 µM) AfAgo concentrations (Fig. 2, Supplementary Fig. S2). Next, to determine the specificity of AfAgo for the terminal bases, we employed a set of ssRNA oligonucleotides with varying 1st, 2nd, and 3rd 5′-terminal nucleotides (Table 1, Fig. 2). AfAgo showed a preference for the 5′-AUU-containing ssRNA (Table 1, Fig. 2 ), while substitution of each of the three 5′-terminal nucleotides of the preferred 5′-AUU ssRNA (1st A, 2nd and 3rd U) reduced the binding affinity (Fig. 2, Table 1). This shows that AfAgo is capable of discriminating the first three 5′-terminal nucleotides of bound ssRNA and has a preference for a 5′-AUU RNA sequence in vitro.

**Table 1:** Apparent K_d_ of different tested nucleic acid substrates determined using EMSA.

Oligonucleotide	5′-terminus	K_d_, nM
ssRNA	AUU	3.8 ± 0.1
	GUU	42 ± 2.1
	CUU	28 ± 2.7
	UUU	15 ± 2.8
	AGU	84 ± 2.9
	AUC	16 ± 2.1
ssDNA	ATT	236 ± 35
RNA/DNA	AUU	6.1 ± 0.04
dsDNA	ATT	37 ± 17
dsRNA	AUU	15 ± 0.9
Nucleic acid binding by AfAgo-gRNA complex		
Guide	Target	K_d_, nM
ssRNA	ssDNA	7.5 ± 0.4
	ssRNA	33 ± 4.2

K_d_ of a pre-formed AfAgo-guide complex with ssRNA and ssDNA target oligonucleotide were determined for one putative optimal ssRNA guide and two targets complementary to the guide within the “seed” region. K_d_ was calculated from experimental results where heparin was omitted. Values are means ± standard deviation of three independent replicates.

**Figure 3:**
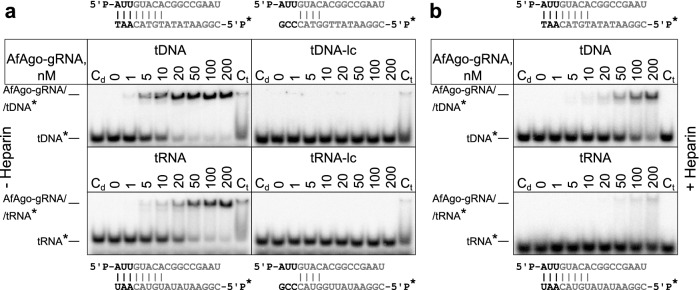
AfAgo RNA-guided NA targeting mechanism and double-stranded nucleic acid binding probed using EMSA. (**a**) Titration of labelled target ssDNA (top) and ssRNA (bottom) with a pre-formed AfAgo-guide RNA complex (1:2 ratio, AfAgo concentrations indicated above each lane) for either 8 nt complementary (left) or 4 nt complementary (lc-“low complementarity”, right) targets. A schematic of guide-target complementarity is shown adjacent to each respective gel, with 5′-terminal bases of the guide and 3′-terminal bases relevant to AfAgo base recognition highlighted in black, remaining strands in grey. 5′^32^P-labelled target strands are denoted with an asterisk. C_d_-duplex control, where guide and target were mixed in the absence of AfAgo at a ratio equivalent to lane “200”. C_t_-target control, where RNA-free AfAgo was mixed with the target at a ratio equivalent to lane “200”. (**b**) Experiment equivalent to (**a**), left, conducted in the presence of 100 ng/µl heparin.

Argonaute proteins usually use a nucleic acid guide to search for and bind a complementary target. Binding experiments described above suggest that AfAgo may use ssRNA guides with 5′-AUU terminal nucleotides. Thus, in the next set of experiments we employed EMSA to test RNA-guided DNA and RNA targeting by AfAgo (Fig. 3). We found that AfAgo pre-loaded with a guide RNA (gRNA) specifically binds DNA and RNA targets, showing higher affinity to DNA targets. To further probe discrimination of DNA *vs.* RNA targets by the AfAgo-gRNA complex, we supplemented the target binding reaction with heparin, a competitor of nucleic acid binding (Fig. 3B). We found that under these conditions AfAgo-gRNA complex shows an even stronger preference for DNA targets over RNA targets, similarly to related long-B and short pAgos^15,16,36^ (Table 1). Experiments with pre-formed RNA/RNA, RNA/DNA and DNA/DNA duplexes (Table 1, Supplementary Fig. S2) were also consistent with the mechanism where AfAgo uses ssRNA as a guide for recognition of ssDNA targets.

### Structural basis for the recognition of the 5′-terminal bases

**Figure 4:**
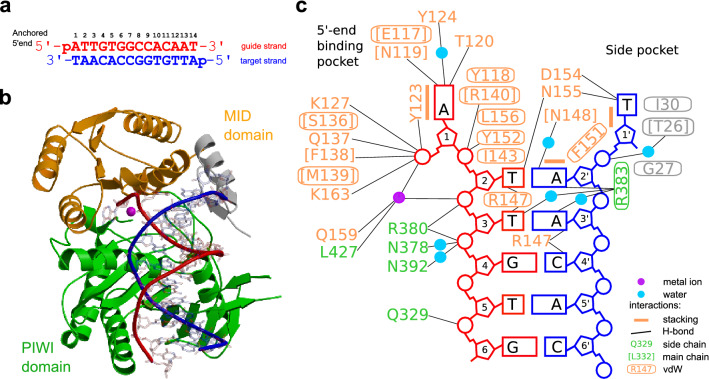
Structure of the AfAgo-DNA complex. (**a**) The 5′-ATT DNA oligoduplex used for crystallization. (**b**) The overall structure of the AfAgo-DNA complex. The backbone of DNA strands is coloured as in A. DNA bases are transparent. The Mg^2+^ ion involved in coordination of the 5′-phosphate of the guide strand is shown as a magenta sphere. (**c**) Schematic representation of AfAgo contacts with the DNA.

To obtain structural insights into the mechanism of 5′-terminus recognition, we have attempted to crystallize AfAgo with various RNA guides carrying 5′-AUU and RNA/DNA heteroduplexes, as well as with various DNA oligoduplexes. Crystallization attempts with RNA-containing duplexes were not successful, but we were able to solve crystal structures of AfAgo in complex with self-complementary 14 bp DNA-DNA oligoduplexes carrying a 5′P-ATT terminus, which is analogous to the optimal ssRNA terminus 5′-AUU (PDB ID 6T5T and 6TUO, respectively) and a 5′P-ATC terminus, which is analogous to a suboptimal ssRNA terminus 5′-AUC (PDB ID 6XUP and 6XU0) (Supplementary Table S2, Fig. 4). Although AfAgo interactions with 5′-ATT DNA oligoduplexes revealed in our structures may not directly translate to possible interactions with 5′-AUU RNA guide, our data provide valuable insights into the base-specific interactions formed by AfAgo and the guide-target duplex.

The AfAgo complex with the suboptimal 5′P-ATC DNA formed crystals of P1 symmetry that contained two DNA-bound protein subunits per asymmetric unit. The main conformations of both subunits are nearly identical, the most significant difference being the main chain conformation of the loop formed by residues 144–149 (Supplementary Fig. S3 A). Electron density for both DNA strands is good only for the first 5–6 bp, i.e. the part that makes direct contacts to the protein; the remaining part of the DNA duplex points into solution and is disordered. The overall structure of AfAgo protein superimposes closely with the previously published AfAgo complexes with RNA and DNA^32,34,35^ (RMSD 1–1.64 Å when overlaid by residues 11–427).

**Figure 5:**
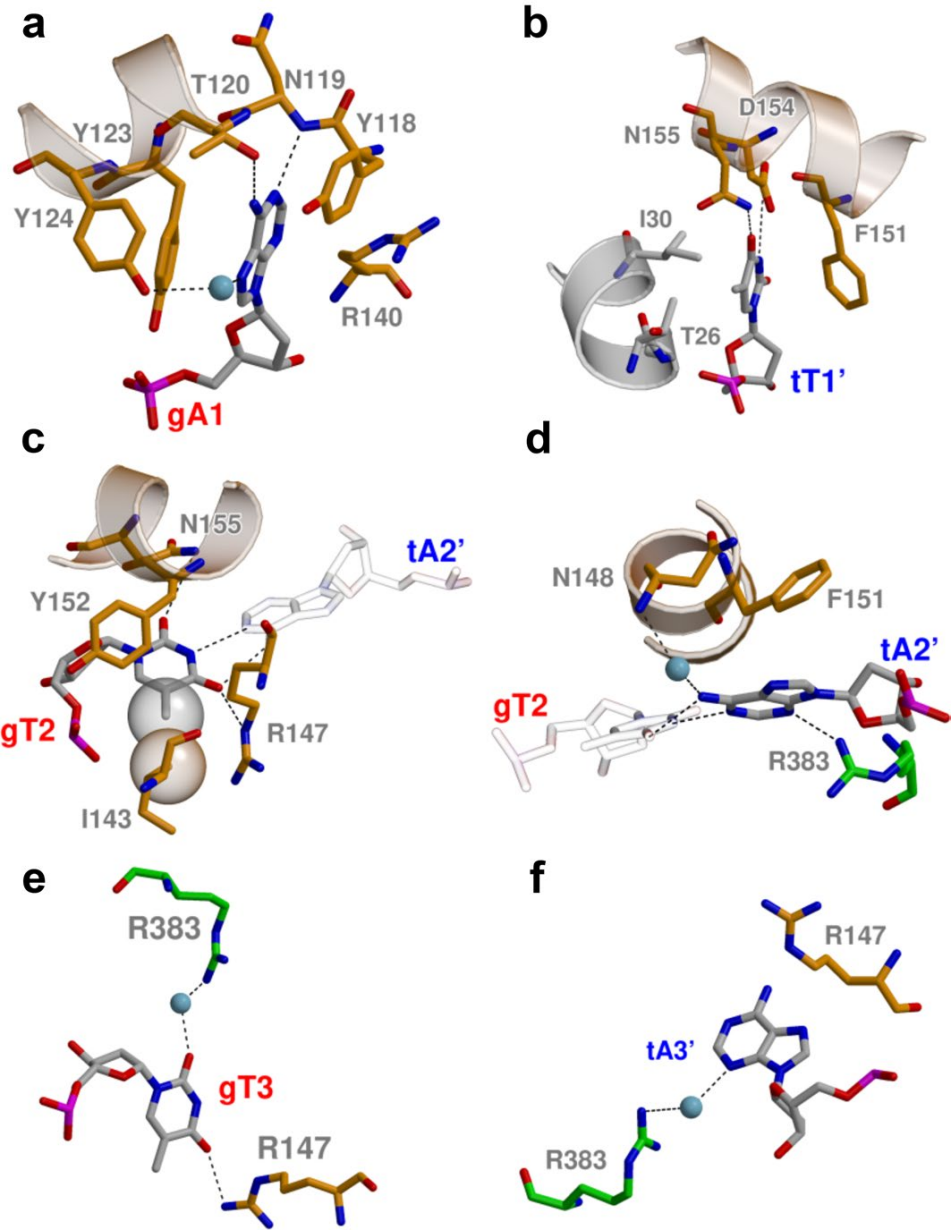
AfAgo interaction with the first three base pairs of the 5′-ATT DNA duplex. gA1 (**a**) and tT1‘ (**b**) in their respective pockets. (**c**, **d**) Recognition of gT2 and tA2’ of the second base pair. (**e**, **f**) Interactions with gT3 and tA3’ of the third base pair.

The AfAgo complexes with two variants of the 5′P-ATT DNA oligoduplex crystallized in the P22_1_2_1_ space group and contained a single DNA-bound protein subunit per asymmetric unit (Supplementary Table S2). The distal end of bound DNA in this crystal form was fixed by crystal packing against the neighbouring protein subunit, thereby helping to model the full-length oligoduplex. The guide strand anchors to AfAgo via its 5′-phosphate group, which is accommodated in the conserved MID domain binding pocket^32,34,35^, where it makes direct contacts with Lys127, Ser136, Gln137, Phe138, Met139 and Lys163, and Mg^2+^-mediated contacts with Gln159 and the C-terminal Leu427 (Fig. 4C). The gA1:tT1’ base pair (the first guide strand adenine and the complementary target strand thymine) is disrupted, with the bases flipped into separate protein pockets. The flipped gA1 base is inserted into the MID domain pocket, where it is fixed by stacking between Tyr123 and Tyr118, base-specific H-bonds to the main chain N atom of Asn119 and the hydroxyl group of Thr120, and a water-mediated H-bond to the hydroxyl of Tyr124 (Figs. 4C, 5A). The tT1’ base of the target strand is displaced into the "side" pocket (Figs. 4C, 5B) formed by helices 26–36 (linker domain) and 149–163 (MID domain), where it stacks against Phe151 and forms H-bonds with Asp154 and Asn155. It should be noted that Asn155 can be modelled in two orientations, one of which is fixed by an H-bond between the OD1 atom to the main chain amide of Phe382. In this case, the ND2 atom of Asn155 is capable of H-bonding to the tT1’ and gT2 bases (Fig. 4).

The base pairs following the gA1:tT1’ of the bound DNA oligoduplex remain undisrupted. As summarized in Extended Data Table 2, AfAgo makes direct contacts to the 2nd and 3rd base pairs. Most extensive sequence-specific contacts are made by the gT2 base of the second base pair, including the contact of the thymine methyl group to Ile143, and base-specific H-bonds of thymine O2 and O4 atoms to Asn155 and Arg147, respectively (Fig. 5C). The tA2’ adenine from the complementary strand stacks against Phe151, and makes a water-mediated H-bond to the main chain of Asn148 from the major groove side and a direct H-bond to Arg383 from the minor groove side (Fig. 5D). Bases from the gT3-tA3' make two water-mediated H-bonds to the protein, Arg383 in the minor groove side, and Arg147 in the major groove side (Fig. 5E,F). In the AfAgo crystal structures with 5′-ATC oligoduplex, we observe two slightly different patterns of the interaction with the 2nd and the 3rd base pairs due to two different conformations of the loop 144–149 that includes residues Arg147 and Asn148 (Supplementary Fig. S3B,C, Extended Data Table 2), one of which is nearly identical to that found in AfAgo crystal structures with 5′-ATT oligoduplexes. Taken together, the structures presented in our work reveal the structural details of AfAgo base-specific interactions with three terminal base pairs of the bound guide/target duplex.

## Discussion

Argonaute proteins use a RNA or DNA guide strand for specific recognition of RNA or DNA target strands^1,13^. Correct base-pairing between the two strands triggers target strand cleavage (catalytically active eAgos and long pAgos involved in antiviral defence) or recruitment of partner proteins (catalytically inactive eAgos). However, the function and action mechanisms of most prokaryotic Ago proteins, in particular catalytically inactive ‘short’ pAgos, remain unknown. In this work, we analyzed guide and target strand preferences of a truncated long-B^8^ prokaryotic Argonaute AfAgo from a hyperthermophilic archaeon *A. fulgidus*, and revealed its sequence specificity to the 5′-terminal nucleotides of the guide strand, and to the complementary fragment of the target strand.

First, we show that AfAgo in vivo tightly interacts with nucleic acids, preferentially short RNA fragments with 5′-terminal AUU sequences. Co-purification of pAgo proteins with RNA was observed before, e.g., for RsAgo, which had a preference for 5′-UY RNA^36^. The tight interaction of AfAgo with RNA seemingly contradicts previous studies^32,34^, in which authors described preferential binding of AfAgo to single- and double-stranded DNA, but not RNA. Presumably, this discrepancy arose due to non-optimal 5′-terminal RNA and DNA sequences (5′-U or 5′-C) used, as we find that in vitro AfAgo also preferentially binds RNA with a 5′-AUU terminus and substitutions at the 5′-terminus reduce affinity (Fig. 2, Table 1). This is a clear indication that AfAgo uses ssRNA as the guide strand and is capable of base-specific interactions with its 5′-terminus.

Further, we demonstrate ssDNA and ssRNA target binding activity in vitro of the AfAgo-gRNA complex, which similarly to many other pAgos^13,18^ displays a notable preference for ssDNA targets over ssRNA. This implies that in vivo AfAgo may also use gRNA to target tDNA. While *A. fulgidus* is a hyperthermophilic organism, most of our experiments were performed at room temperature (25 °C, which is not uncommon in the field^37,38^). However, this should not invalidate our conclusions related to AfAgo preferences for ssRNA and ssDNA as the optimal guide and target strands, respectively. Indeed, pre-incubation the AfAgo-ssRNA binding reaction mixtures at elevated temperatures (70 °C) prior to EMSA did not alter the ability of AfAgo to discriminate the 5′-terminal ssRNA sequences, albeit it decreased the observed AfAgo binding affinities to all ssRNA variants (Supplementary Fig. S7), presumably due to the lack of chaperones and other protein-stabilizing factors normally present in host cells.

We have also solved four crystal structures of AfAgo bound to DNA-DNA oligoduplexes with the 5′-AT terminal sequences, which mimic the 5′-AU terminus of the in vivo bound RNAs. Two structures were obtained with different optimal-like 5′P-ATT oligoduplexes (PDB ID 6T5T and 6TUO, respectively) and two structures with a suboptimal-like 5′-ATC oligoduplex (PDB ID 6XUP and 6XU0). Although interactions with 5′-ATT oligoduplexes may not directly translate to possible interactions with 5′-AUU RNA guides, where AfAgo might adopt a slightly different conformation and potentially interact with the RNA guide less strongly, our structural data suggest that AfAgo employs base-specific readout of the terminal nucleotides of the bound guide and target strands (Figs. 4, 5, Extended Data Table 2). This interpretation is consistent with the previously published structures of AfAgo bound to the non-optimal DNA duplexes (5′P-TTC, PDB ID 2W42^35^ and 5′P-UUC, PDB ID 2BGG^32^), and to the near-optimal RNA-RNA duplex (5′-terminal sequence 5′P-AGA, PDB ID 1YTU^34^).

The most extensive base-specific contacts are made to the 5′-terminal guide strand adenine (gA1) and the complementary target strand thymine tT1’, which is disrupted, and the bases are placed into separate protein pockets (Extended Data Table 2). As shown in Supplementary Fig. S4, interactions with gA1 observed in our structures with duplex DNA are very similar to those observed in the RNA-bound structure 1YTU^34^ (bases at other positions of these structures differ and therefore can not be directly compared). This similarity of base-specific contacts observed with gRNA^34^ and DNA [this work] indicates that the terminus of the gDNA/tDNA duplex used in our study may provide an adequate mimic for the optimal gRNA/tDNA heteroduplex. Similar disruption of the equivalent base pair was also observed in the AfAgo structures with non-optimal 5′-terminal nucleotides (PDB ID 2W42^35^ and 2BGG^32^). In this case, the flipped gT/U1 base in the 5′-end binding pocket is unable to form adenine-specific contacts observed in our crystal structures, including H-bonds with gA1 base made by Asn119 main chain N, Thr120 OH and water-mediated H-bond between 124 OH group and N7 atom of gA1 (Supplementary Fig. S5). Since in all available structures of AfAgo with RNA the unpaired t1' base does not enter the "side" pocket, we can only compare the "side" pocket interactions of tT1' in our structures with tA1' in PDB ID 2W42^35^ (Supplementary Fig. S5B). In our structures the Asn155 side chain interacts simultaneously with both t1' (tT1’) and g2 (gT2) bases, and tT1' makes an additional H-bond with Asp154. In 2W42^35^, the tA1’ base in the "side" pocket makes an H-bond with the side chain of Asp154, but the conformation of Asn155 is not suitable for interaction with tA1'. Base-specific contacts formed by the bases of the second guide strand nucleotide gT2 and its complementary target strand nucleotide tA2’ are less numerous (Fig. 5, Extended Data Table 2), but still sufficient for discrimination against alternative base pairs.

Specific recognition of both guide and target strand nucleotides distinguishes AfAgo from previously characterized Argonaute proteins, which limit specific recognition of terminal nucleotides either to the guide strand (e.g., RsAgo, PDB ID 6D8P^29^), or to the target strand^39^. Another unique feature of AfAgo is that it is a homodimeric protein that can bring together two copies of the guide-target duplex^40^. In crystal structures presented in this study we observed the same dimerization mode (in 6T5T and 6TUO dimer is formed by a crystallographic symmetry operator), raising further questions regarding possible AfAgo functions in vivo. To understand its role in bacterial cells, we are currently performing further structural and functional studies of AfAgo and its putative partner proteins encoded in the same operon.

## Materials and methods

### Nucleic acids used

All nucleic acids used in this work were supplied by Metabion.

### Proteins and their complexes with nucleic acids

*E. coli* cells expressing His_6_-tagged AfAgo were disrupted by sonication in a lysis buffer containing 20 mM Tris–HCl (pH 8.0 at 25 °C), 500 mM NaCl, 5 mM 2-mercaptoethanol, supplemented with 2 mM phenylmethylsulfonyl fluoride, incubated for 20 min at 50 °C; cell debris was removed by centrifugation at 48,400 × g for 1 h. The supernatant was loaded onto a HiTrap chelating HP column charged with Ni^2+^ (GE Healthcare) and eluted with a linear gradient (15–500 mM) of imidazole in the lysis buffer. Protein-containing fractions were pooled, diluted to 0.2 M of NaCl with a buffer containing 20 mM Tris–HCl (pH 8.0 at 25 °C), 10% (v/v) glycerol, 5 mM 2-mercaptoethanol and incubated for 1 h at 37 °C with 1 mM EDTA (ethylenediaminetetraacetic acid) and RNase A/T1 (ThermoFisher Scientific) (1:100). Next, the protein solution was centrifuged at 48,400 g for 30 min, the supernatant containing RNA-free AfAgo was loaded onto a HiTrap Heparin HP column (GE Healthcare) and eluted using a 0.2–1.0 M NaCl gradient. Finally, the protein was run through the HiLoad 16/600 Superdex 200 column (GE Healthcare) in lysis buffer supplemented with NaCl to 1 M and dialyzed against 20 mM Tris–HCl (pH 8.0 at 25 °C), 500 mM NaCl, 50% (v/v) glycerol. AfAgo with bound RNA was purified as above, omitting RNase treatment.

### RNA sequencing and analysis

The pETDuet-1 plasmid with the cloned hypothetical protein AfAgo from *Archaeoglobus fulgidus* DSM 4304 (GenBank accession nos. NP_070147.1 and NC_000917.1, respectively) was used in this work. AfAgo-bound RNA was purified using phenol isolation from an RNase-untreated AfAgo preparation. RNA sequencing was performed as described in^41^. The raw reads were first processed by trimming adapter sequences using AdapterRemoval^42^. Reads then were aligned to the reference genome with BWA-MEM^43^. After the alignment, only the aligned reads were retrieved from the alignment file using the ‘bam2fastq’ program from the SAMtools toolkit^44^. The processed reads were analysed using a Unix ‘awk’ filter to extract RNA sequences and a Perl program that counted the occurrence of each RNA base in the first 50 positions of the reads. The weblogo^45^ program was used to plot these nucleotide frequencies. The most frequently occurring nucleotide letter in the first position was A (86.2%) and in the second position was U (84.6%); these positions were also among the most reliable positions in all reads.

The raw RNA reads are deposited to the Sequence Read Archive as the file “LT_02.fq.bz2” (sample name “LT_02”) under the BioProject accession number PRJNA763829.

### EMSA experiments

Nucleic acid-free AfAgo was diluted to a 2 × final concentration in a binding buffer consisting of 40 mM Tris–acetate (pH 8.4 at 25 °C), 1 mM EDTA, 100 mM potassium acetate (KAc), 0.1 mg/ml bovine serum albumin, 1 mM DTT and 10% (v/v) glycerol, and mixed in different ratios with 5′^32^P-labelled nucleic acids, also pre-diluted to a 2 × final concentration in the same buffer. Nucleic acids used for binding studies were MZ-1480 and MZ-1698-1708 for ssRNA and MZ-1447 for ssDNA. Duplexes were prepared by annealing MZ-1480 to MZ-1481 for dsRNA, MZ-1447 to MZ-1455 for dsDNA, and MZ-1480 to MZ-1455 for RNA/DNA heteroduplex. Nucleic acids were diluted such that the final NA-protein mixture would contain 1 nM 5′^32^P-labelled and 4 nM unlabelled 5′P-NA. The binding reaction mixture was incubated for 10 min at room temperature (25 °C) and loaded onto an 8% PAAG gel (29:1 acrylamide/bis-acrylamide) prepared with 40 mM Tris–acetate (pH 8.4 at 25 °C), 1 mM EDTA, 100 mM KAc. Additional experiments with MZ-1480 and MZ-1707 were conducted by incubating the binding reaction mixture for 10 min at 70 °C. Electrophoresis was run at room temperature in all cases. To study the RNA-guided nucleic acid targeting mechanism, AfAgo was pre-mixed with MZ-1480, a 5′P-ssRNA guide, at 0.4:0.8 µM ratio of AfAgo:guide, incubated for 10 min at room temperature and diluted to 2 × final binding reaction concentration in the same buffer as above. Diluted target NAs were added to the reaction mixture at a 1:1 volumetric ratio to a final reaction concentration of 5 nM (1 nM 5′^32^P + 4 nM 5′P), the mixture was incubated for 10 min at room temperature and analyzed as described above. Target NAs used were MZ-1556 and MZ-1557 as 8- and 4-nucleotide RNA targets, respectively, and MZ-1560 and MZ-1561 as analogous DNA targets, respectively. For the heparin-supplemented reactions, heparin was pre-mixed with the target NAs before adding them to the binding reaction mixture so that the final heparin concentration was 100 ng/µl. Radiolabelled substrates were detected and quantified using a phosphor imager. The results were analysed with OptiQuant and OriginPro software. The K_d_ was calculated from the following formula:

$$S_{NB} = A1 + \frac{{\frac{100}{{S_{0} }}\left( {S_{0} \frac{100 - A1}{{100}} - E_{0} - K_{d} + \sqrt {\left( {S_{0} \frac{100 - A1}{{100}} + E_{0} + K_{d} } \right)^{2} - 4S_{0} E_{0} \frac{100 - A1}{{100}}} } \right)}}{2}.$$

where S_NB_ unbound substrate, nM; S_0_ initial substrate concentration, nM; E_0_ initial protein complex concentration, nM; K_d_ dissociation constant, A1 nonbinding fraction of substrate, %.

### Crystallization and structure determination

AfAgo used for crystallization was pre-treated with RNase A/T1 Mix (ThermoFisher Scientific): 2 µl of RNase Mix was added to 2 ml of 1.9 mg/ml AfAgo in a storage buffer and incubated for 30 min at ambient temperature. Complexes of AfAgo with DNA were prepared by mixing protein solution in a storage buffer with an equimolar amount of oligoduplex in the presence of 2 mM DTT and 5 mM MgCl_2_. Glycerol was removed using NAP columns (GE Healthcare) equilibrated with 20 mM Tris–HCl (pH 7.5 at 25 °C), 150 mM NaCl, 5 mM MgCl_2_ and 2 mM DTT. Complexes were concentrated by ultrafiltration. The concentration of the complexes used in crystallization trials was in the range of 90–120 µM (as monomer). Crystallization experiments were prepared by mixing the protein solution with equal volumes of crystallization buffers (Supplementary Table S2) in sitting drops. Crystals were grown in a cold room (4–8 °C). Prior to flash cryo-cooling to 100 K, crystals were washed in the cryo-protection buffers (Supplementary Table S2).

Four datasets were collected at EMBL P14 and P13 beamlines on the PETRA III ring of DESY synchrotron in Hamburg (Germany. The datasets were processed by XDS^46^ and by CCP4 software^47^. The structures were solved by molecular replacement using MOLREP v11.6.04^48^ with PDB entries 1YTU and 2W42 as models. Structures were refined with REFMAC v5.8.0230^49^ and PHENIX v1.13^50^ and remodelled using COOT v0.8.9.1^51^. The data collection and refinement statistics are presented in Supplementary Table S2. The representative electron density maps for the AfAgo interactions with gA1 and tT1' bases are presented in the Supplementary Fig. S6.

## Supplementary Information

Supplementary Information 1.

Supplementary Information 2.

Supplementary Information 3.

## Data Availability

Cited: *Archaeoglobus fulgidus* DSM 4304 genomic sequence, GenBank accession no. NC_000917.1. Protein AfAgo, GenBank accession no. NP_070147.1. AfAgo complexed with a 16 nt DNA duplex, PDB ID: 2W42. AfAgo complexed with 21 nt RNA duplex, PDB ID: 1YTU. Ternary RsAgo complex containing guide RNA paired with target DNA, PDB ID: 6D8P. Obtained in this work: Raw RNA sequencing reads from this study, BioProject accession number PRJNA763829, https://www.ncbi.nlm.nih.gov/bioproject/763829. AfAgo in complex with dsDNA, PDB IDs: 6T5T, 6TUO, 6XUP, 6XU0.
