# Supplementary material for: Structural basis for sequence-specific recognition of guide and target strands by the Archaeoglobus fulgidus Argonaute protein
Source: Sci Rep. 2023 Apr 14;13:6123. doi: 10.1038/s41598-023-32600-w (PMC10104839; doi:10.1038/s41598-023-32600-w)

Structural basis for sequence-specific recognition of guide and target strands by the *Archaeoglobus fulgidus* Argonaute protein

Elena Manakova[#], Edvardas Golovinas[#], Reda Pocevičiūtė, Giedrius Sasnauskas, Algirdas Grybauskas, Saulius Gražulis, Mindaugas Zaremba[*]

Institute of Biotechnology, Life Sciences Center, Vilnius University, Sauletekio av. 7, LT-10257, Vilnius, Lithuania.

*To whom correspondence should be addressed. Tel: +370-5-2234357; Fax: +370-5-2234367; Email: zare@ibt.lt.

[#]These authors contributed equally: Elena Manakova, Edvardas Golovinas.

SUPPLEMENTARY INFORMATION

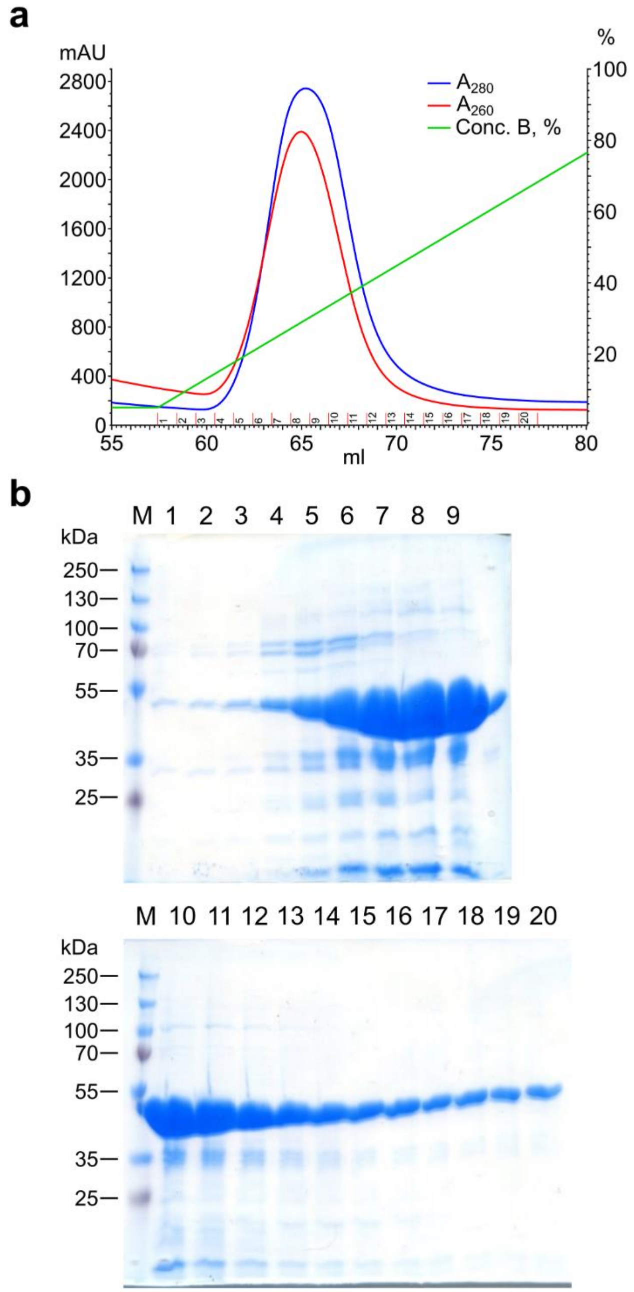

Supplementary Figure S1. Purification of AfAgo through a HisTrap affinity column. (**a**) Chromatogram. A_{280} – blue; A_{260} – red; percentile fraction of elution buffer B – green. Collected fractions are indicated below the curves. (**b**) SDS-PAGE of fractions collected; numbering corresponds to numbers in (**a**). M – mass marker.

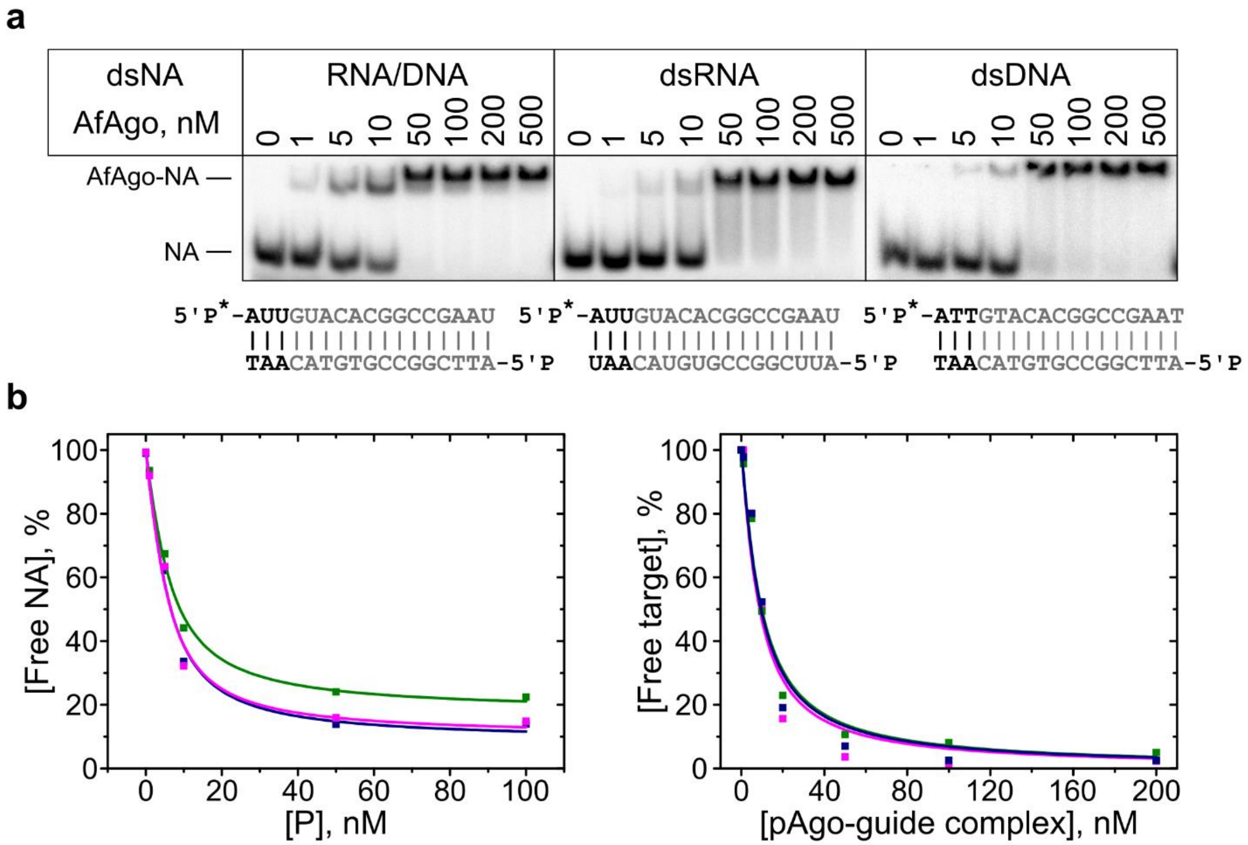

Supplementary Figure S2. (**a**) Double-stranded nucleic acid binding by AfAgo studied by EMSA. AfAgo concentrations are indicated above each lane. Double-stranded nucleic acid sequences and structure are depicted schematically below each gel, with 5′-terminal bases of the guide and 3′-terminal bases relevant to AfAgo base recognition highlighted in black, remaining strands in grey. 5′^{32}P-labelled strand indicated with an asterisk. (**b**) Representative binding fit curves of several independent replicates used to calculate K_d of ssRNA guide binding by AfAgo (left) and of target DNA binding by AfAgo-gRNA complex (right).

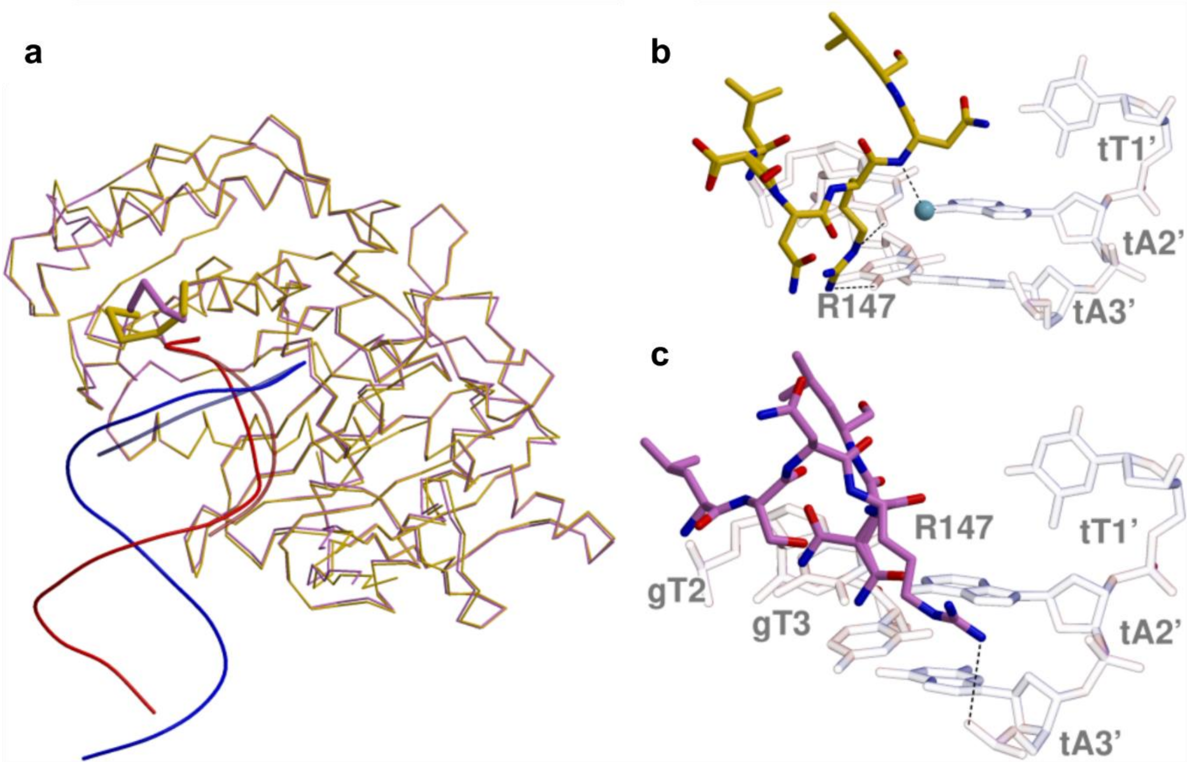

Supplementary Figure S3. Different conformation of the loop 144-149 in crystal structures of AfAgo. (**a**) AfAgo protein chains from the complex with 5′-ATT (PDB ID **6T5T**, yellow) and with 5′-ATC (PDB ID **6XUP chain A**, magenta) are shown as traces. Guide and target DNA strands are red and blue, respectively. (**b**) The conformation of the loop 144-149 in the structure AfAgo-5′ATT, PDB ID **6T5T**. (**c**) Loop 144-149 from the A protein chain in the crystal structure AfAgo-5′ATC, PDB ID **6XUP**.

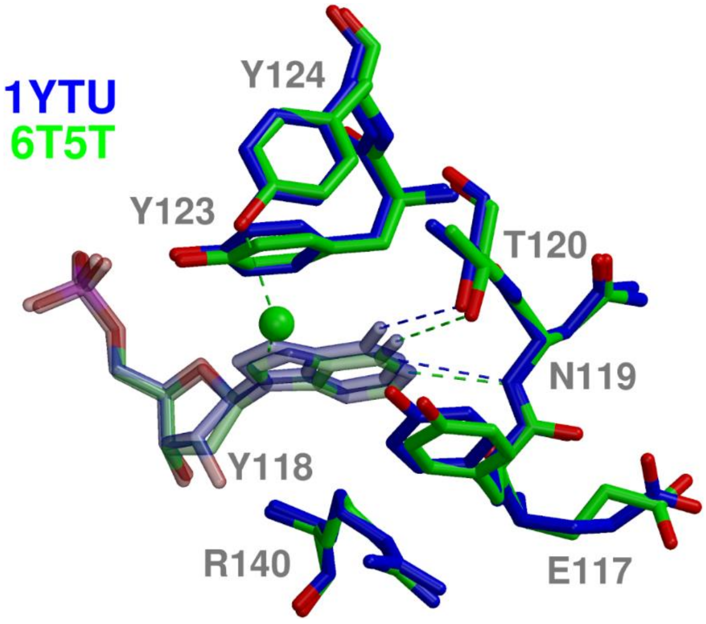

Supplementary Figure S4. Binding of g1A base in 6T5T and 1YTU. The water molecule from 6T5T is shown as a green sphere. H-bonds are shown as dashed lines.

a

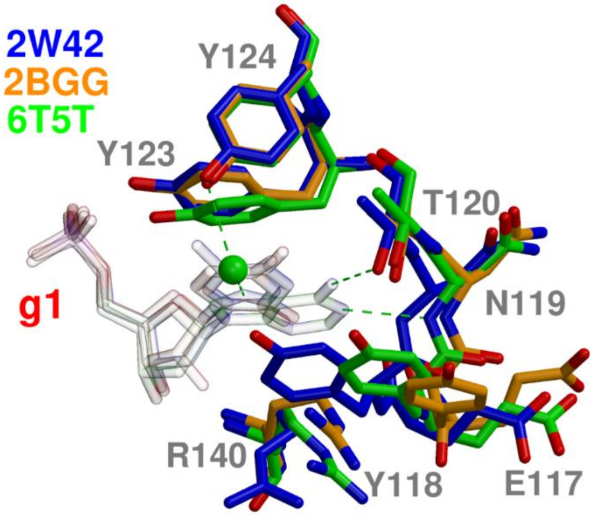

b

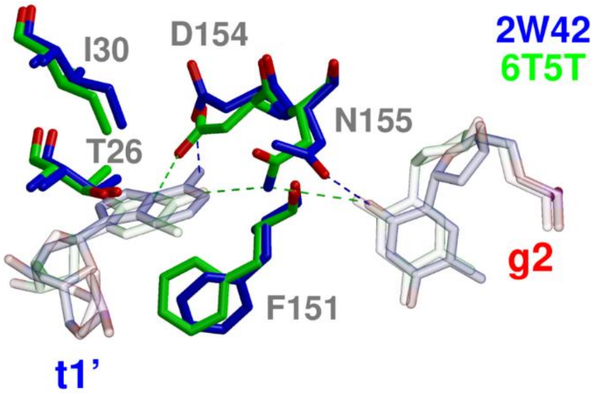

Supplementary Figure S5. (**a)**, Comparison of the first guide base in crystal structures of AfAgo 2W42, 2BGG and 6T5T. (**b)**, Binding of gT2 and t1' bases in the "side" pocket in 2W42 and 6T5T.

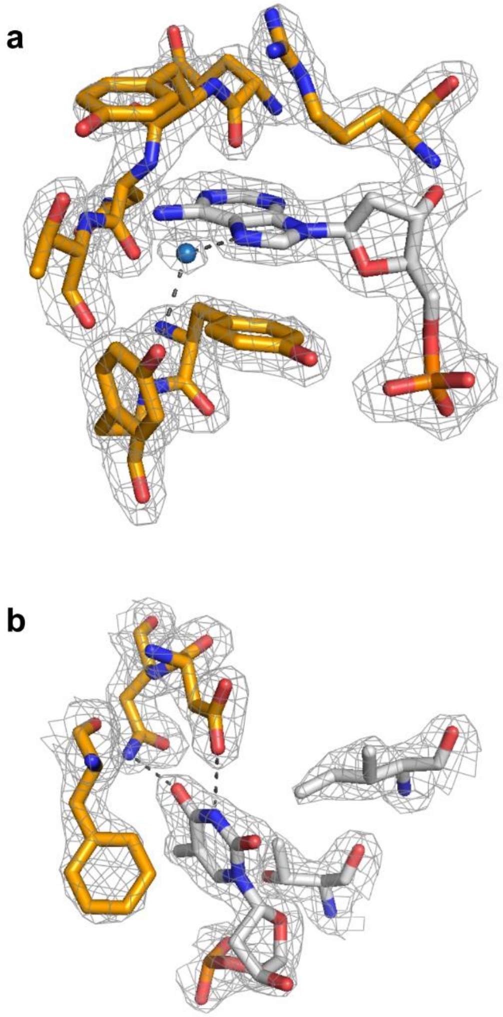

Supplementary Figure S6. The electron density is $2F_o$-F_c at 1.6 sigma. (**a)**, gA1 base recognition. (**b**), tT1’ base recognition.

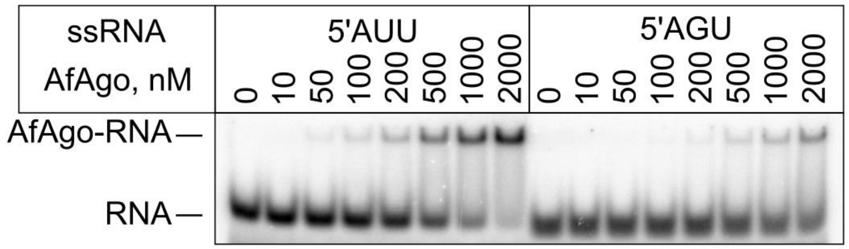

Supplementary Figure S7. AfAgo interactions with ssRNA *in vitro* at elevated temperature. EMSA experiments were performed with 5 nM total 5′P-ssRNA, complexes pre-formed at 70 °C, gels were run at room temperature. The general binding affinity of ssRNAs decreased, however the specificity of AfAgo towards a 5′AUU over 5′AGU ssRNA remains.

Supplementary Table S1. Oligonucleotides used in this work.

Name	Sequence, 5′->3′	Application
MZ-952	ATCGTGGCCACGAT	Crystallization; self-complementary
MZ-1288	ATTGTGGCCACAAT	Crystallization; self-complementary
MZ-1289	ATTGTACGTACAAT	Crystallization; self-complementary
MZ-1447	ATTGTACACGGCCGAAT	ssDNA for EMSA
MZ-1455	ATTCGGCCGTGTACAAT	Duplex formation with MZ-1447 and MZ-1480 for EMSA
MZ-1480	AUUGUACACGGCCGAAU	ssRNA for EMSA and gRNA for RNA-guided NA targeting experiments
MZ-1481	AUUCGGCCGUGUACAAU	Duplex formation with MZ-1480 for EMSA
MZ-1556	CGGAAUAUAUGUACAAU	8 nt complementary target RNA
MZ-1557	CGGAAUAUUGGUACCCG	4 nt complementary target RNA
MZ-1560	CGGAATATATGTACAAT	8 nt complementary target DNA
MZ-1561	CGGAATATTGGTACCCG	4 nt complementary target DNA
MZ-1698	GUUGUACACGGCCGAAC	ssRNA for EMSA
MZ-1699	UUUGUACACGGCCGAAA	ssRNA for EMSA
MZ-1706	AUCGUACACGGCCGGAU	ssRNA for EMSA
MZ-1707	AGUGUACACGGCCGACU	ssRNA for EMSA
MZ-1708	CUUGUACACGGCCGAAG	ssRNA for EMSA

Supplementary table S2. Data collection and refinement statistics.

Oligoduplex	**ATC**GTGGCCAC**GAT**<br>||||||||||||||| **TAG**CACCGGTG**CTA**	**ATC**GTGGCCAC**GAT**<br>||||||||||||||| **TAG**CACCGGTG**CTA**	**ATT**GTGGCCAC**AAT**<br>||||||||||||||| **TAA**CACCGGTG**TTA**	**ATT**GTACGTAC**AAT**<br>||||||||||||||| **TAA**CATGCATG**TTA**
Crystallization buffer	50 mM sodium cacodylate (pH 5.5 at 25 °C), 120 mM KCl, 10 mM $MgCl_2$, 7% (w/v) PEG3350, 5% (v/v) glycerol	50 mM sodium cacodylate (pH 6.5 at 25 °C), 40 mM KCl, 10 mM $MgCl_2$, 11% (w/v) PEG3350	50 mM sodium cacodylate (pH 5.5 at 25 °C), 200 mM KCl, 10 mM $MgCl_2$, 5% (w/v) PEG4000, 5% (v/v) glycerol	
Cryo protection buffer	100 mM sodium cacodylate (pH 5.5 at 25 °C), 200 mM KCl, 10 mM $MgCl_2$, 20% PEG3350 (w/v), 10% (v/v) glycerol			100 mM sodium cacodylate (pH 6.5 at 25 °C), 40 mM KCl, 10 mM $MgCl_2$, 20% (w/v) PEG3350, 20% (v/v) glycerol
Data collection statistics				
Space group	P 1	P 1	P 2 21 21	P 2 21 21
Cell constants a, b, c, α, β, γ	a=51.80 Å, b=60.87 Å, c=101.72 Å, α=76.56°, β=75.59°, γ=79.39°	a=51.91 Å, b=61.20 Å, c=103.09 Å, α=98.32°, β=104.96°, γ=100.62°	a=52.10 Å, b=99.55 Å, c=109.90 Å, α=β=γ=90°	a=52.01 Å, b=99.63 Å, c=109.80 Å, α=β=γ=90°
Wavelength, Å	0.97630	0.97630	0.97970	0.97970
X-ray source	PETRA III, EMBL C/O DESY, P14	PETRA III, EMBL C/O DESY, P14	PETRA III, EMBL C/O DESY, P13	PETRA III, EMBL C/O DESY, P13
Unique reflections: overall (outer shell)	83713 (4556)	85105 (12355)	63695 (9192)	53664 (3128)

Resolution range, Å	41.50 - 1.90	54.90 - 1.80	99.52 - 1.70	99.63 - 1.80
Completeness: overall (outer shell), %	91.6 (91.2)	91.5 (90.9)	100 (100)	99.9 (100)
Multiplicity: overall (outer shell)	3.8 (3.9)	3.9 (3.9)	10.3 (10.1)	6.5 (6.7)
I/σ: overall (outer shell)	11.2 (1.6)	8.3 (1.7)	20.8 (2.3)	20.8 (2.6)
Rmerge: overall (outer shell), %	4.9 (82.1)	6.1 (63.8)	6.3 (96.9)	4.4 (74.8)
B-factor from Wilson, $Å^2$	36.4	35.0	30.8	29.6
Refinement statistics				
Resolution range, Å	40.72 - 1.90	46.45 - 1.90	54.95 - 1.70	54.90 - 1.80
Reflections: work (non-anomalous)/test	83692 (9394)	85025 (8390)	63601 (6249)	53695 (5011)
Atom number: protein/solvent	7671 (449)	7175 (495)	4325 (349)	4325 (394)
Rcryst (Rfree), %	18.1 (22.4)	17.7 (22.2)	18.3 (21.6)	18.8 (23.1)
RMSD: bond lengths, Å / bond angles, (°)	0.010 / 0.999	0.012 / 1.104	0.012 / 1.148	0.005 / 0.753
Ramachandran: favoured/allowed/	96.3 / 3.7 / 0	96.6 / 3.4 / 0	98 / 8 / 0	97.24 / 2.51 / 0.25

outliers, %				
Average B-factors:<br><br>all atoms/ main chain/ side chain/ solvent/ DNA, Å^2	48.0 / 42.5 / 43.9 / 53.0 / 81.2	46.9 / 40.0 / 40.5 / 49.2 / 85.9	42.0 / 33.8 / 39.6 / 46.0 / 74.2	47.0 / 35.3 / 41.3 / 48.6 / 91.1
PDB ID	6XUP	6XU0	6T5T	6TUO

SOURCE DATA

Source data Figure 1A

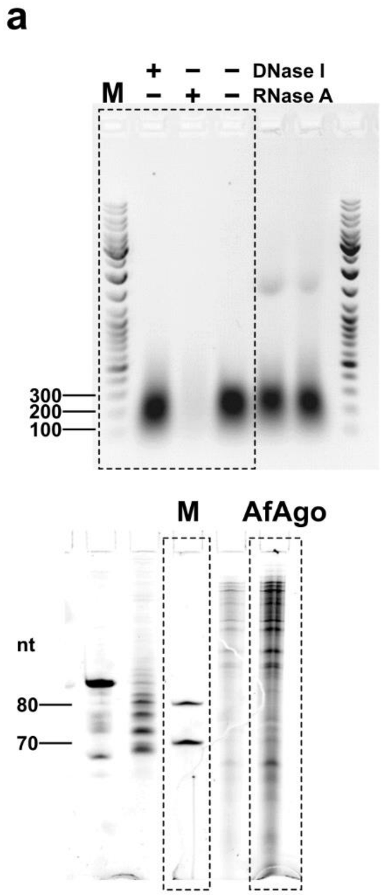

Source Data Figure 2A

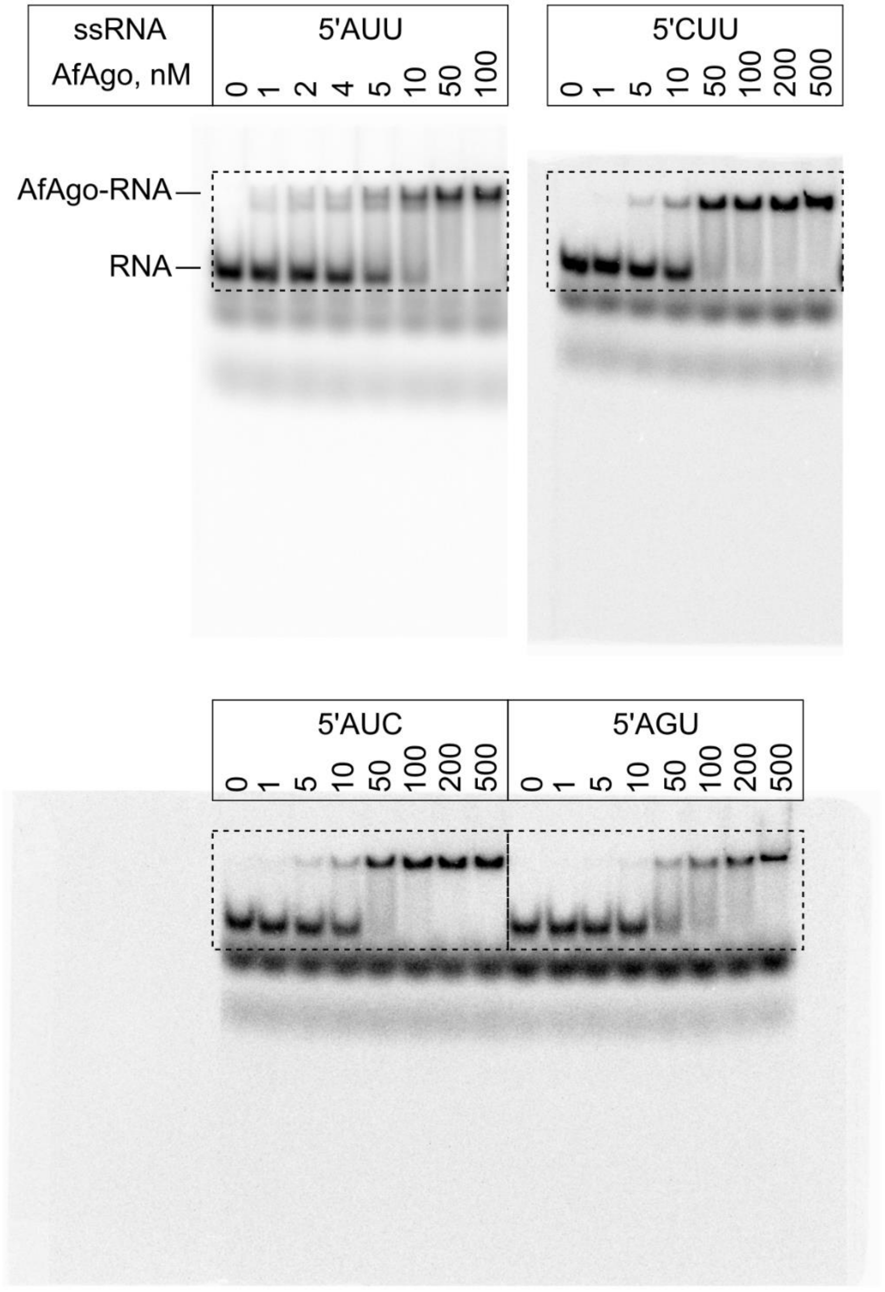

Source data Figure 2A

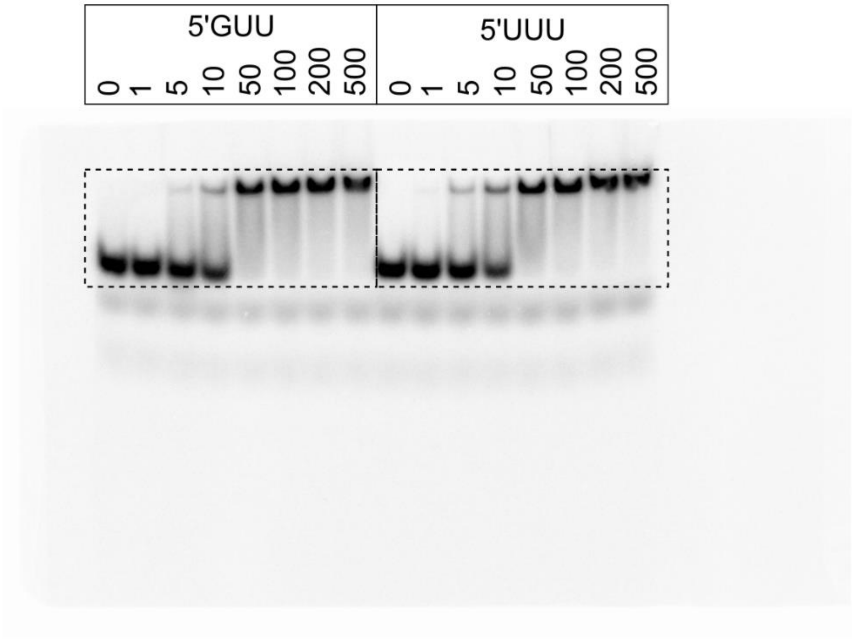

Source data Figure 2B

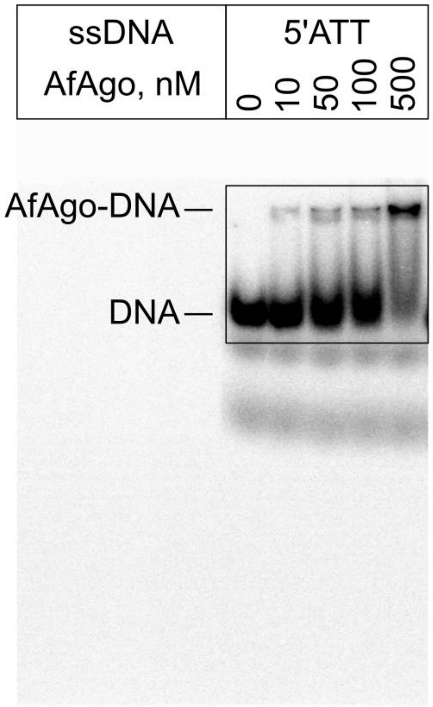

Source data Figure 3A

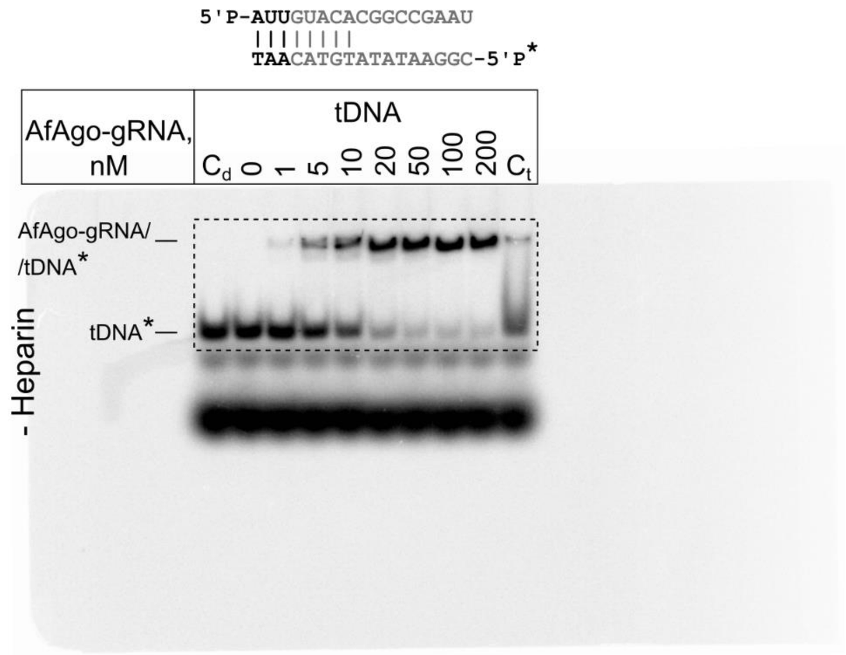

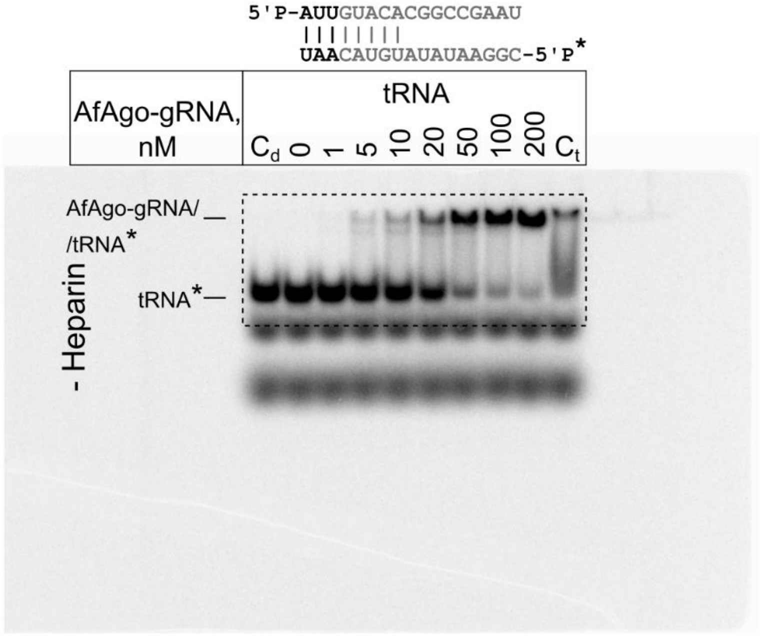

Source data Figure 3A

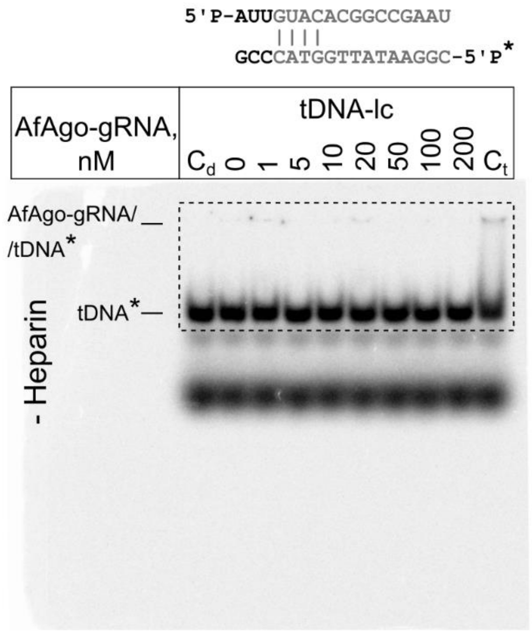

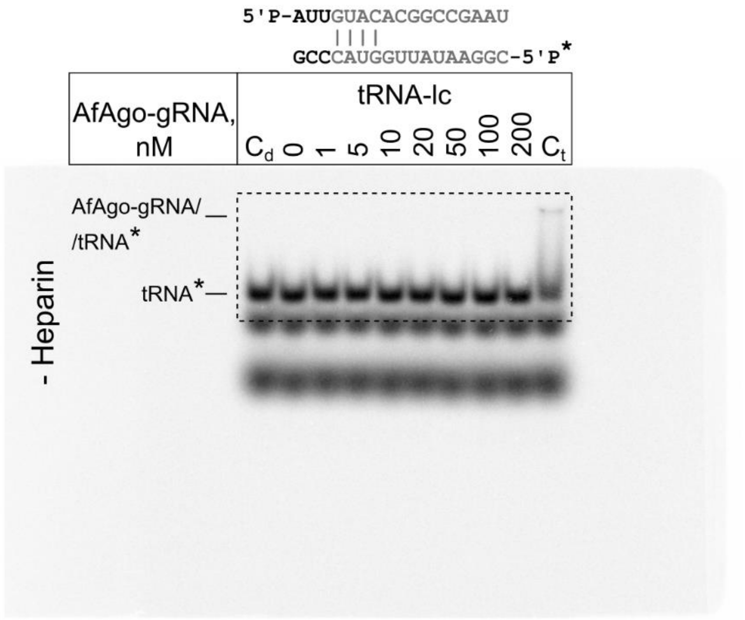

Source data Figure 3B

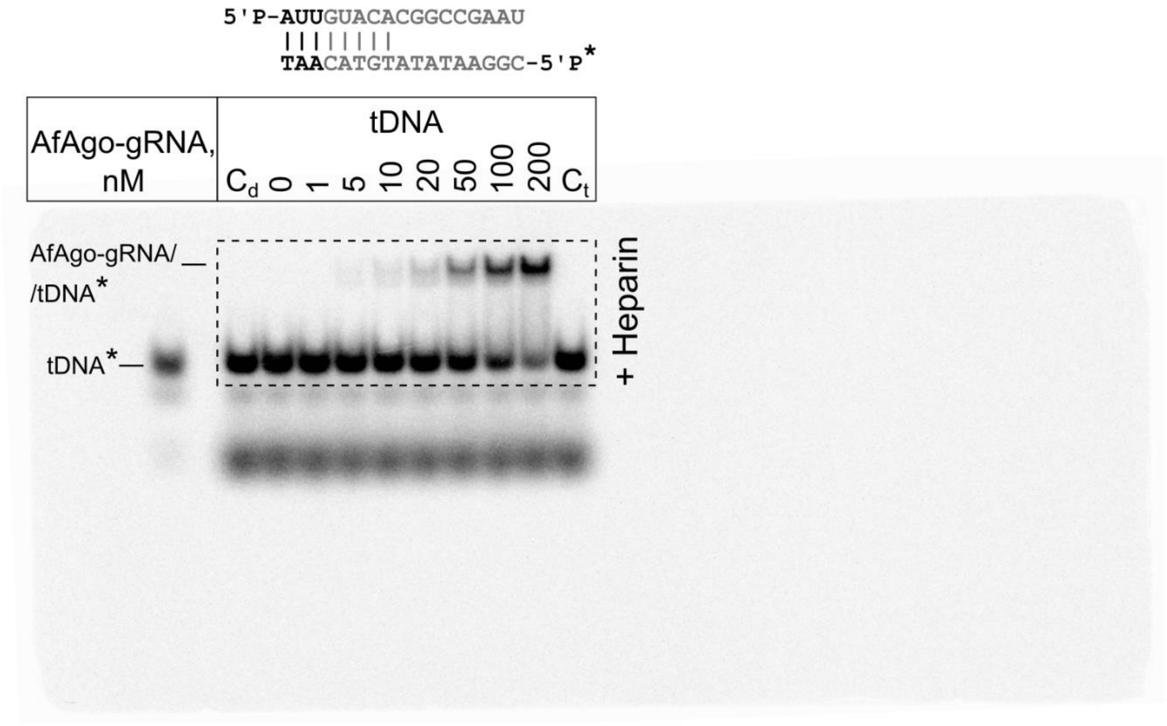

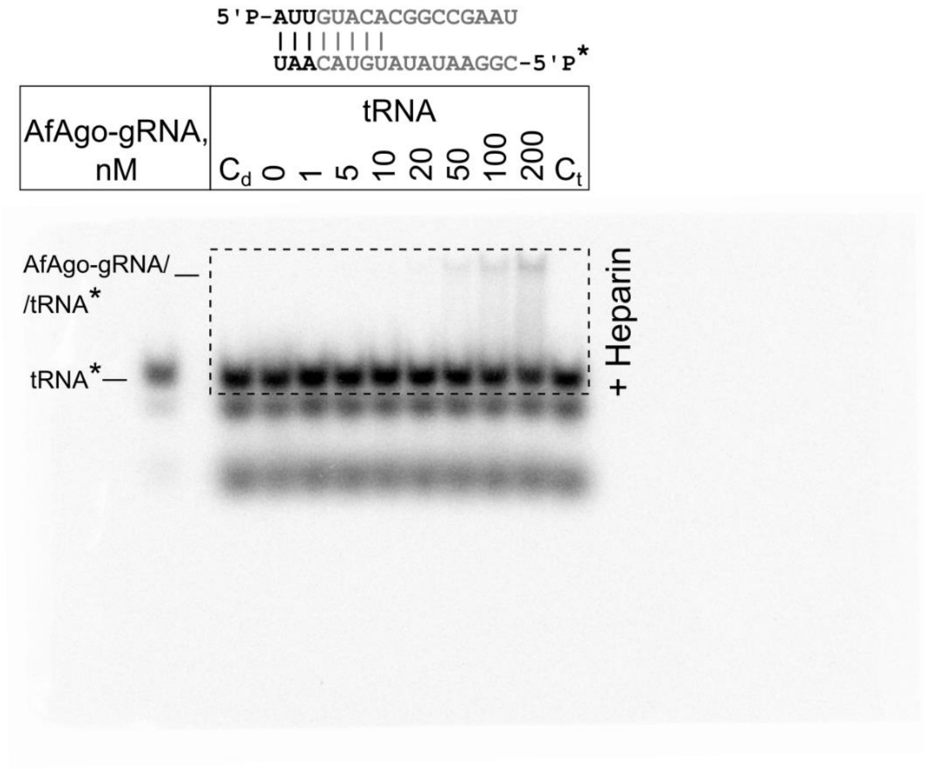

Source data Figure S2A

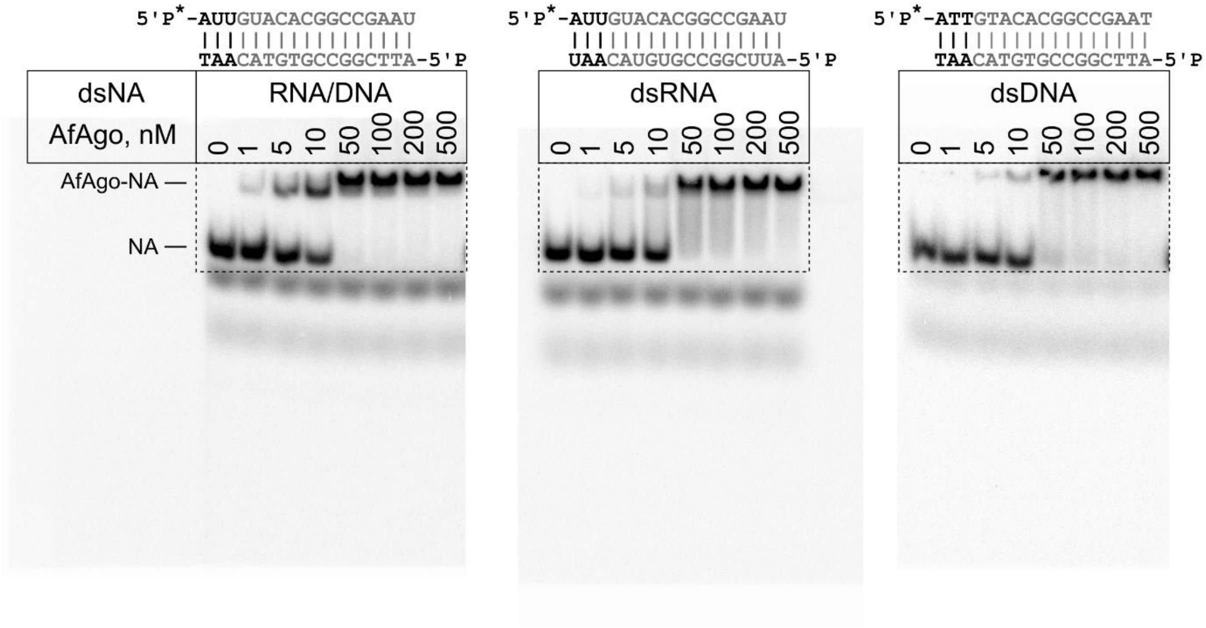

Source data Figure S7

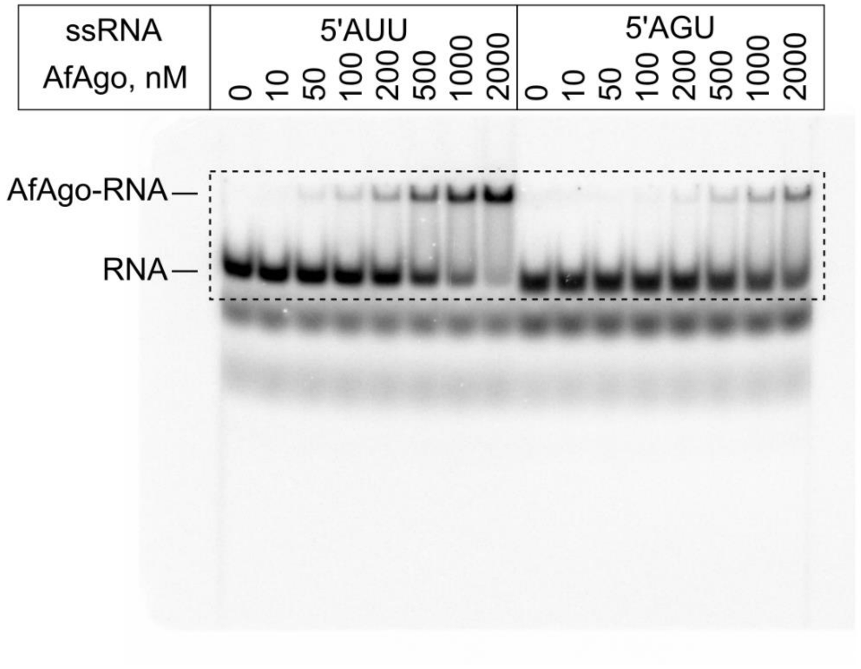

Supplement: Supplementary file 2 — Supplementary Information 2. [file 41598_2023_32600_MOESM2_ESM.pdf]
